# *Streptomyces natalensis* programmed cell death and morphological differentiation are dependent on oxidative stress

**DOI:** 10.1038/srep12887

**Published:** 2015-08-10

**Authors:** Tiago Beites, Paulo Oliveira, Beatriz Rioseras, Sílvia D. S. Pires, Rute Oliveira, Paula Tamagnini, Pedro Moradas-Ferreira, Ángel Manteca, Marta V. Mendes

**Affiliations:** 1 i3S–Instituto de Investigação e Inovação em Saúde, Universidade do Porto, Porto, Portugal; 2 IBMC—Instituto de Biologia Molecular e Celular, Universidade do Porto, Porto, Portugal; 3 Área de Microbiología, Departamento de Biología Funcional e IUOPA, Facultad de Medicina, Universidad de Oviedo, Oviedo, Spain; 4 ICBAS—Instituto de Ciências Biomédicas Abel Salazar, Universidade do Porto, Porto, Portugal; 5 Faculdade de Ciências, Departamento de Biologia, Universidade do Porto, Porto, Portugal

## Abstract

*Streptomyces* are aerobic Gram-positive bacteria characterized by a complex life cycle that includes hyphae differentiation and spore formation. Morphological differentiation is triggered by stressful conditions and takes place in a pro-oxidant environment, which sets the basis for an involvement of the oxidative stress response in this cellular process. Characterization of the phenotypic traits of *Streptomyces natalensis* Δ*katA1* (mono-functional catalase) and Δ*catR* (Fur-like repressor of *katA1* expression) strains in solid medium revealed that both mutants had an impaired morphological development process. The sub-lethal oxidative stress caused by the absence of KatA1 resulted in the formation of a highly proliferative and undifferentiated vegetative mycelium, whereas de-repression of CatR regulon, from which KatA1 is the only known representative, resulted in the formation of scarce aerial mycelium. Both mutant strains had the transcription of genes associated with aerial mycelium formation and biosynthesis of the hyphae hydrophobic layer down-regulated. The first round of the programmed cell death (PCD) was inhibited in both strains which caused the prevalence of the transient primary mycelium (MI) over secondary mycelium (MII). Our data shows that the first round of PCD and morphological differentiation in *S. natalensis* is dependent on oxidative stress in the right amount at the right time.

*Streptomyces* is the largest genus of the Actinobacteria phylum, which constitutes a very robust phylogenetic group of Gram-positive bacteria with high G + C content^1^. *Streptomyces* display an aerobic lifestyle and undergo a complex life cycle that comprises hyphae differentiation and formation of resistant unigenomic spores^2^. Furthermore, streptomycetes are most noticeable for their ability to produce a plethora of secondary metabolites with a wide array of biological activities, e.g. antifungals, anticancer agents or immunosuppressants^3^.

During the last decade, several studies have demonstrated that the *Streptomyces* typical life cycle on solid medium is more complex than initially thought. A previously unsuspected highly compartmentalized mycelium was shown to be formed after spore germination. This primary mycelium (MI) displays an active primary metabolism and undergoes an early event of programmed cell death (PCD) at certain hyphae segments^4^,^5^. A multinucleated secondary mycelium (MII) arises from the viable segments of MI and starts to grow into and above the solid medium. The passage into this developmental stage is accompanied with the metabolic shift from primary to secondary metabolism and with the triggering of the development program^5^,^6^. As a consequence, biosynthetic gene clusters of secondary metabolites are activated and proteins involved in the onset of aerial mycelium are expressed, namely the components of the hydrophobic layer: chaplins, rodlins and the lantipeptide SapB^5^. In parallel with aerial mycelium formation, the innermost mycelium suffers a second round of PCD, possibly to increase nutrient availability^6^. Finally, spores are formed through septation of aerial hyphae.

The development program in streptomycetes is orchestrated by BldD that avoids a premature morphological differentiation by repressing the transcription of many development related genes in a manner that depends on cyclic di-GMP-mediated dimerization^7^,^8^. Among BldD targets, BldH and BldN play a pivotal role in aerial mycelium formation since they activate the transcription of genes coding for the lantipeptide SapB^9^,^10^, chaplins (Chp) and rodlins (Rdl)^11^.

Aerial mycelium formation occurs in an environment with a high oxygen pressure, which promotes the generation of reactive oxygen species (ROS). Interestingly, it has been demonstrated that ROS may display multiple functions in the development of different microorganisms^12^. Moreover, the development program in streptomycetes is triggered by environmental stressful conditions (e.g. nutrient scarcity) that are usually accompanied with oxidative stress^13^,^14^,^15^. Thus, although largely uncomprehended, there are evidences pointing out to an active involvement of oxidative stress in *Streptomyces* development.

*Streptomyces* possess a fine-tuned oxidative stress response, which likely evolved as a response to its aerobic lifestyle. Typically, streptomycetes possess several H_2_O_2_-sensitive transcription regulators, namely CatR (Fur-like), OxyR (LysR-family) and the sigma/anti-sigma factors SigR/RsrA that control the expression of the catalase KatA1, the alkylhydroperoxide reductase system AhpCD and the thioredoxin system TrxAB, respectively^16^,^17^,^18^; an organic hydroperoxide-sensitive transcription regulator OhrR (MarR-family) that controls the organic peroxide resistance proteins OhrABC^19^; and the nickel-responsive transcription regulator Nur (Fur-like) that governs the expression of iron- and nickel-superoxide dismutases SodF and SodN^20^.

Previously we have shown that the deletion of genes encoding enzymes involved in the oxidative stress response, namely the alkylhydroperoxide reductase system (*ahpCD*), the mono-functional catalase (*katA1*) and the superoxide anion scavenging enzyme superoxide dismutase (*sodF*), led to an effective modulation of intracellular ROS levels in *Streptomyces natalensis* that resulted in a modulation of secondary metabolism in liquid medium^21^. Unlike the other mutant strains, Δ*katA1* presented an impaired aerial mycelium formation, i.e. it displayed a bald phenotype. In this work we generated a *S. natalensis* defective mutant on the *katA1* transcription repressor encoding gene *catR* that displayed a severe, but not complete, impairment of aerial mycelium formation. Characterization of *S. natalensis* Δ*catR* and Δ*katA1* strains on solid medium provided, for the first time, evidences regarding the influence of oxidative stress over *Streptomyces* morphological differentiation.

## Results

### Genes associated with morphological development and oxidative stress response in *S. natalensis* ATCC 27448

The *S. natalensis* ATCC 27448 genome sequence (GenBank Accession Number: JRKI01000000) was analysed for the presence of genes associated with *Streptomyces* morphological differentiation (reviewed in Claessen, *et al*.^22^, Flardh and Buttner^2^ and McCormick and Flardh^23^). Orthologues for the most important development genes of the model organism *S. coelicolor* are present in the *S. natalensis* genome (summarized in Supplementary Table S1).

*S. natalensis* ATCC 27448 harbours homologues to genes related to the formation of aerial mycelium (e.g. *bldD*, *bldH* and *bldN*), biosynthesis of the aerial hyphae hydrophobic layer, cell division and chromosome segregation (e.g. *whiA*, *whiB* and *ftsZ*) and spore maturation (e.g. the *whiE* locus). Regarding proteins related to aerial mycelium hydrophobic layer, *S. natalensis* possesses two long chaplins (SNA_06310 and SNA_01235), seven short chaplins (SNA_00490, SNA_12255, SNA_11245, SNA_11250, SNA_00485, SNA_05975 and SNA_12040) and one rodlin encoding gene (SNA_27905), differing from *S. coelicolor* that presents three long chaplins, five short-chaplins and two rodlin encoding genes. *S. natalensis* lantipeptide SapB biosynthetic genes (*ram* genes) are arranged into a cluster, similarly to what is observed in *S. coelicolor* and other streptomycetes. Finally, it is noteworthy that the *whiE* locus coding for the grey polyketide spore pigment in *S. natalensis* displays a high degree of synteny with its *S. coelicolor* counterpart.

Regarding genes associated with the oxidative stress response, particularly those related to H_2_O_2_ detoxification, in addition to the previously described H_2_O_2_-inducible mono-functional catalase *katA1* (SNA_34325), its Fur-like repressor *catR* (SNA_34330), the alkyl hydroperoxide reductase system (*ahpCD*, SNA_35115 and SNA_35120) and its H_2_O_2_ responsive regulator *oxyR* (SNA_35110)^21^, we have identified an additional Clade 3 mono-functional catalase *katA3* (SNA_29075) and a bi-functional catalase-peroxidase encoding gene *cpx* (SNA_19710) in *S. natalensis*.

### The knock-out mutants for catalase and Fur-like repressor CatR present impaired aerial mycelium formation

In a previous study, we generated a *S. natalensis* mutant defective on the mono-functional catalase KatA1 that displayed an impaired morphological differentiation in solid medium^21^. To investigate the effect of KatA1 activity in the development program of *S. natalensis*, we further analysed the Δ*katA1* strain and a knock-out mutant on the *katA1* transcription repressor, CatR. *S. natalensis* Δ*catR*::*aac(3)IV-oriT* mutant strain was generated by conjugation using a PCR targeting procedure that replaced the native *catR* locus with an apramycin resistance cassette. Several exconjugants were obtained that displayed the same phenotype. Three exconjugants were randomly selected and their identity confirmed by PCR and Southern blot hybridization; one exconjugant was randomly selected for subsequent studies and named *S. natalensis* Δ*catR* (Supplementary Fig. S1).

**Figure 1:**
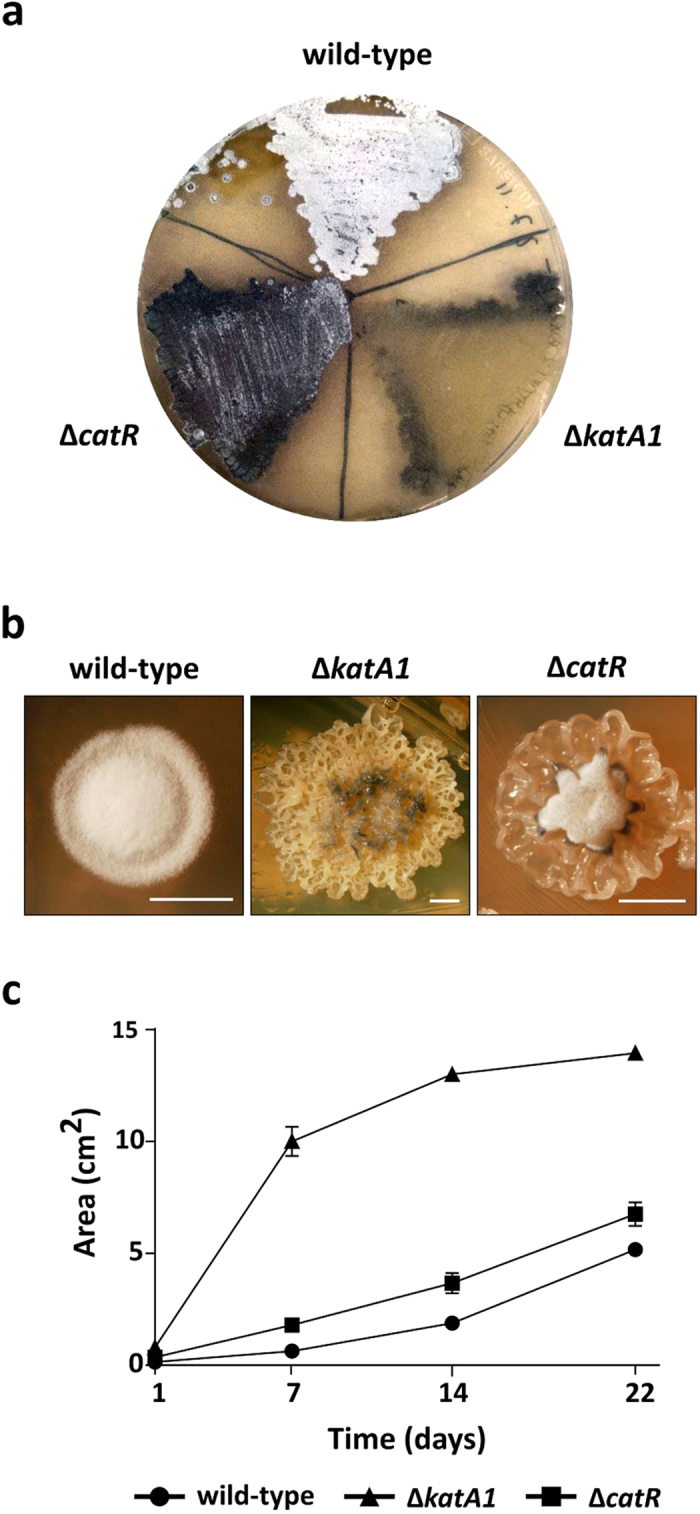
Morphological phenotypes and mycelium proliferation assay of *S. natalensis* wild-type, Δ*katA1* and Δ*catR* strains grown in R5 solid medium. (**a**) Photographs of mycelium lawns at 72 h. (**b**) Photographs of isolated colonies at 72 h. Scale bar: 2,50 mm. Photographs by Tiago Beites. (**c**) Mycelium proliferation rate in R5 solid medium. 10 μl drops of liquid cultures grown to an OD_600nm_ of 4–5 were placed in the middle of R5 plates and mycelium proliferation was measured for 22 days. R5 plates were photographed and the mycelium area was determined using the measure function of the ImageJ software. Results are representative of three independent experiments.

Morphological development of the *S. natalensis* wild-type, Δ*katA1* and Δ*catR* strains was characterized in R5 medium growing as mycelium lawns and as isolated colonies (Fig. 1). In the wild-type mycelium lawn, aerial mycelium was first observed at 48 h after inoculation (data not shown) and at 72 h was fully formed (Fig. 1A). Δ*catR* strain development was delayed when compared to the wild-type forming a scarce aerial mycelium at 72 h, which did not further develop in subsequent days (data not shown). Moreover, Δ*catR* accumulated a dark-blue pigment that might correspond to the blue-coloured pigment previously observed in *S. natalensis* Δ*sngA* liquid cultures^24^. Δ*katA1* did not form aerial mycelium at any time point, i.e. it displayed a bald phenotype (Fig. 1A). The phenotypes of isolated colonies were similar to those observed in mycelium lawns. However, isolated colonies allowed us to notice that the mutant strains vegetative mycelium presented a higher growth rate, leading to the formation of larger colonies than in the wild-type strain (notice the differences in the scale bars in Fig. 1B). This was particularly visible in Δ*katA1*, which completely lacked the typical wild-type colony structure (Fig. 1B).

To characterize the vegetative mycelium growth rate, we assessed the rate of mycelium proliferation (Fig. 1C). With this purpose, a 10 μl drop of YEME liquid cultures grown to an OD_600nm_ of 4–5 was inoculated in the centre of R5 plates and mycelium proliferation was followed for 22 days by measuring the vegetative mycelium area. Both mutant strains presented a higher rate of mycelium proliferation when compared with the wild-type, in particular Δ*katA1* strain that at day 22 occupied a 2.7-fold larger area than the wild-type (Fig. 1C).

### Key development genes are down-regulated in Δ*katA1* and Δ*catR*

**Figure 2:**
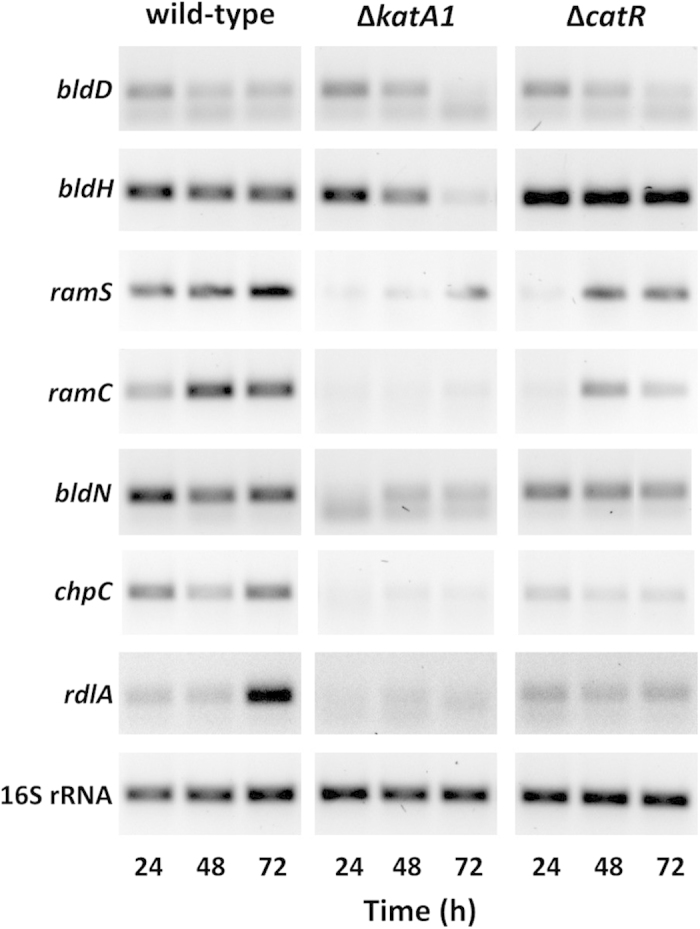
Transcription analysis by RT-PCR of genes associated with the development program in *S. natalensis* wild-type, Δ*katA1* and Δ*catR* strains. The used pair of primers and annealing temperatures are presented in Supplementary Table S2. The amplicons are the result of 30 PCR cycles. Transcription profiles are representative of three independent experiments.

To further characterize the morphological impairment phenotype displayed by the mutant strains in solid medium we examined the transcription of genes associated with the onset of aerial mycelium (*bldD*, *bldH* and *bldN*) and formation of the aerial hyphae hydrophobic coat (*ramS*, *ramC*, *chpC*, and *rdlA*) at 24 h (vegetative growth), 48 h and 72 h (formation of aerial mycelium) (Fig. 2). No major differences were observed for *bldD* expression under the conditions tested. However, Δ*katA1* strain displayed a progressive decrease of *bldH* transcript levels in contrast with the wild-type and Δ*catR* strains that presented a constitutive expression. Interestingly, the transcription of *ramC* and *ramS* was down-regulated in both mutant strains when compared to the wild-type, pointing out to a deficient SapB production, particularly in Δ*katA1* strain. In addition, the poor correlation between the transcription of *bldH* and *ram* genes, especially in Δ*catR* strain, suggests the presence of additional players in the regulation of SapB biosynthesis in *S. natalensis*.

The transcription of *bldN*, *chpC* and *rdlA* was down-regulated in Δ*katA1* and, to a lesser extent, in Δ*catR* when compared to the wild-type. These results suggest a defective synthesis of chaplins and rodlins in the mutant strains.

### The oxidative stress defences are activated at early developmental stages in Δ*katA1* and Δ*catR*

**Figure 3:**
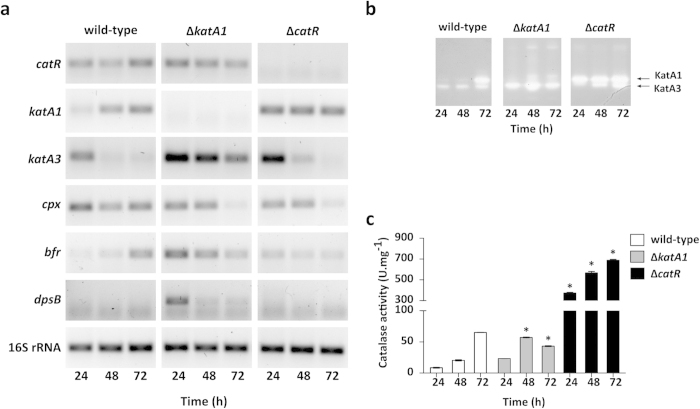
Characterization of oxidative stress response in *S. natalensis* wild-type, Δ*katA1* and Δ*catR* strains. (**a**) Transcription analysis of genes associated with the oxidative stress response by RT-PCR. The used pair of primers and annealing temperatures are presented in Supplementary Table S2. The amplicons are the result of 30 PCR cycles. The transcription profiles are representative of three independent experiments. (**b**) Catalase activity determined by native-PAGE. 50 μg of total protein per lane were loaded and separated by electrophoresis before in-gel zymography. Arrows indicate bands displaying enzymatic activity. (**c**) Total catalase activity in protein crude extracts. Results (average of triplicates and standard deviation) are representative of three independent experiments. Statistically significant differences to the wild-type strain at each time point were determined by one-way ANOVA followed by post hoc test (Tukey test; GraphPad Prism). * p < 0.01.

We also examined the transcription of genes associated with the oxidative stress response in particular the catalase encoding genes (*katA1*, *katA3* and *cpx*) and the Fur-like repressor *catR* at 24 h (vegetative mycelium), 48 h and 72 h (formation of aerial mycelium) (Fig. 3A).

The wild-type strain displayed a growth stage-dependent transcription of the two mono-functional catalases: while *katA3* was preferably expressed before the onset of aerial hyphae (24 h), *katA1* transcription was temporally correlated with aerial mycelium formation (48 h and 72 h). The increasing expression of KatA1 reflected in a progressive increase in the KatA1 activity band as assessed by native-PAGE (Fig. 3B) and total catalase activity (Fig. 3C). In Δ*katA1* strain*, katA3* transcription was up-regulated at all time points when compared to the wild-type (Fig. 3A). This profile was reflected in a high intensity of KatA3 activity band (Fig. 3B) and a peak at 48 h of total catalase activity levels (Fig. 3C). Regarding Δ*catR* strain, mutated in the repressor of *katA1* (*catR*), the constitutive over-expression of *katA1* (Fig. 3A) and the consequent induction of KatA1 activity (Fig. 3B,C) was an expected outcome. Regarding the transcription pattern of the catalase-peroxidase encoding gene *cpx*, there was no clear correlation with morphological development. Nevertheless, both mutants presented a down-regulation of *cpx* transcription at 72 h.

Toxicity of H_2_O_2_ is intimately associated to the availability of free ferrous iron (Fe^2 + ^) due to the formation of hydroxyl radicals in the so-called Fenton reaction. Iron storage proteins such as bacterioferritins and Dps, play an important role on the oxidative stress response due to their ability to chelate intracellular ferrous iron and circumvent free iron-driven intracellular toxicity^25^. The *in silico* analysis of *S. natalensis* genome revealed two proteins harbouring a ferritin domain (Pfam domain PF00210), SNA_09350 and SNA_32055, orthologues to the *S. coelicolor* bacterioferritin Bfr and DpsB protein, respectively. Analysis of *bfr* expression revealed a clear up-regulation in Δ*katA1* at 24 h and 48 h. In addition, Δ*katA1* was the only strain in which transcripts of *dpsB* could be detected under the conditions tested (24 h).

These results show that Δ*katA1* and Δ*catR* mutant strains have elements of the oxidative stress response system induced at earlier stages of the development program when compared to the wild-type. The expression of iron storage proteins in *S. natalensis* Δ*katA1* indicates that KatA3 activity is not sufficient to counteract the lack of KatA1 concerning H_2_O_2_ detoxification and it is safe to assume that Δ*katA1* endures a sub-lethal oxidative stress. Regarding Δ*catR* strain, induction of catalase activity is the consequence of *catR* deletion and the consequent derepression of *katA1* transcription.

### Δ*katA1* and Δ*catR* presented an extended primary metabolism and inhibition of development-related PCD

Total protein extracts of the wild-type, Δ*katA1* and Δ*catR* strains grown in solid medium for 72 h were analysed by 2D-PAGE (Supplementary Fig. S2). Analysis of the 2D gels revealed a total of 353 valid protein spots. The mutant strains Δ*katA1* and Δ*catR* presented 61 and 33 spots with significant differences when compared with the wild-type, respectively (*P* > 0.05; two-fold change). From these, 41 well-individualized spots were analysed by mass spectrometry (PMF + MSMS). Excluding the spots that were identified as isoforms of the same protein and spots with mixture of proteins, we have successfully identified 26 individual proteins (Table 1; Supplementary Fig. S2).

The set of over-expressed proteins in Δ*katA1* when compared to the wild-type, was majorly associated with energy metabolism (SNA_07865, SNA_09040, SNA_10650, SNA_12565 and SNA_33550) and anabolic processes, such as the biosynthesis of co-factors (SNA_36475), amino acids (SNA_33095) and nucleotides (SNA_12580, SNA_13390 and SNA_36490), as well as protein synthesis (SNA_38815). These findings suggest that, at 72 h, Δ*katA1* maintained active metabolic pathways associated with anabolic processes and energy production. Although at a less extent, we have also identified over-expressed proteins in Δ*catR* related with energy metabolism (SNA_09035, SNA_09040 and SNA_33550).

The set of proteins identified in the wild-type strain that were down-regulated or even not detected in the Δ*katA1* and Δ*catR* protein extracts included the molecular chaperon DnaK (SNA_03365), the proline catabolism enzyme pyrroline-5-carboxylate dehydrogenase (SNA_33060), the pentose phosphate pathway (PPP) enzyme ribokinase (SNA_09155) and the polyribonucleotide nucleotidyltransferase PNPase (SNA_32175). Also, it is noteworthy the absence of PNPase in the mutant strains proteomes; this nuclease was previously shown to be expressed during the developmentally related programmed cell death (PCD) process in *S. coelicolor*^26^.

Finally, stress-related over-expressed proteins were also identified in the protein extracts of Δ*katA1* and Δ*catR* namely the organic hydroperoxide resistance reductase B (SNA_07730) in both mutant strains.

**Figure 4:**
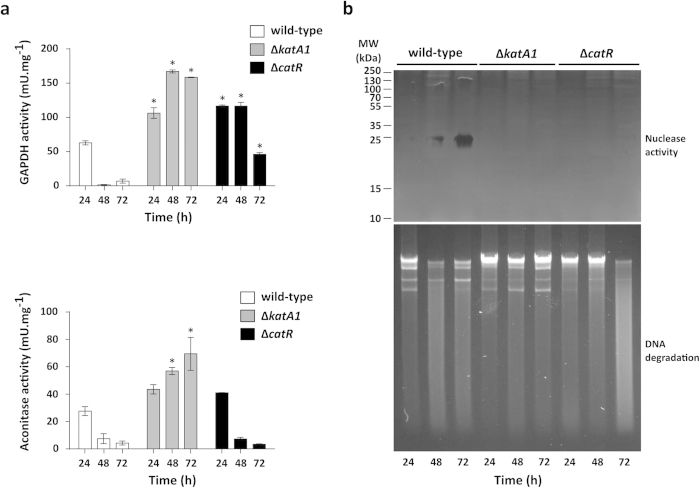
Assessment of primary metabolism enzyme activities and programmed cell death markers in *S. natalensis* wild-type, Δ*katA1* and Δ*catR* strains. (**a**) Determination of GAPDH (top panel) and aconitase (bottom panel) enzymatic activities in protein crude extracts. Results (average of triplicates and standard deviation) are representative of three independent experiments. Statistically significant differences between strains at each time point were assessed by one-way ANOVA followed by post hoc test (Tukey test; GraphPad Prism). * p < 0.01. (**b**) The upper panel depicts nuclease activity in protein crude extracts (20 μg of protein per lane); the SDS-PAGE performed with the protein extracts was used as loading control (Supplementary Fig. S3). The lower panel shows the integrity of total genomic DNA in a 0.8% (w/v) agarose gel (1 μg of total DNA per lane).

Overall, this proteome analysis suggested that the mutant strains, particularly Δ*katA1* strain, displayed an active primary metabolism at 72 h and that the PCD process was inhibited. To further confirm these results we determined the enzymatic activities of central carbon metabolism proteins throughout growth in solid medium, namely the glycolytic enzyme glyceraldehyde 3-phosphate dehydrogenase (GAPDH) and the tricarboxylic acid (TCA) cycle enzyme aconitase (Acn) (Fig. 4A). In addition, we have also characterized two PCD markers: intracellular nuclease activity and DNA degradation (Fig. 4B).

The GAPDH and Acn activity profiles in the wild-type presented a pronounced decrease from 24 h to 48 h, which overlapped with the onset of aerial hyphae formation (Fig. 4A). Conversely, the enzymatic activities of GAPDH and Acn in Δ*katA1* protein crude extracts were significantly higher than the wild-type throughout growth (Fig. 4A). This profile further supports the idea that Δ*katA1* experiences a highly active primary metabolism throughout growth. Finally, *S. natalensis* Δ*catR* presented significantly higher GAPDH activity levels when compared with the wild-type during all growth stages, albeit a down-shift on its activity was also observed upon aerial mycelium emergence (72 h). Acn activity profile was similar to that of the wild-type (Fig. 4A).

Regarding PCD markers, results have shown that the wild-type protein extracts displayed a single nuclease activity band (approx. 25 kDa) with increasing intensity throughout time (Fig. 4B). Noteworthy, *S. coelicolor* protein extracts display a nuclease activity band associated with PCD that presents a similar molecular weight^26^. No nuclease activity was detected in the mutant strains protein extracts (Fig. 4B). Accordingly, DNA was degraded at 48 h and 72 h in the wild-type whilst DNA integrity was maintained throughout growth in the Δ*katA1*. For Δ*catR* strain, DNA degradation was detected at 72 h (Fig. 4B).

### Δ*katA1* development is arrested at MI stage and Δ*catR* presents an extended life-span of MI

**Figure 5:**
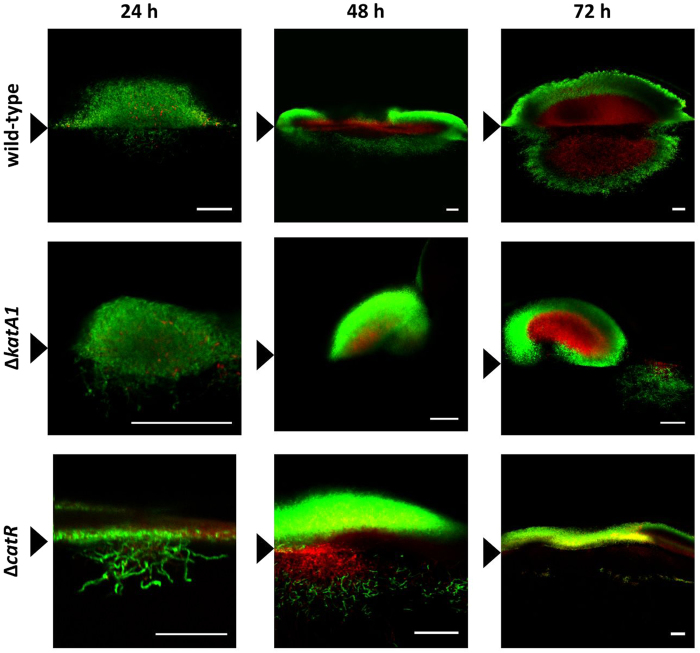
*S. natalensis* wild-type, Δ*katA1* and Δ*catR* strains colony development by confocal microscopy and using a cell viability assay. The presented micrographs are the result of merging the red (dead cells) and green (live cells) channels with ImageJ software. Black arrowheads indicate the medium-air interface. The results are representative of three independent experiments. Scale bar: 40 μm.

**Figure 6:**
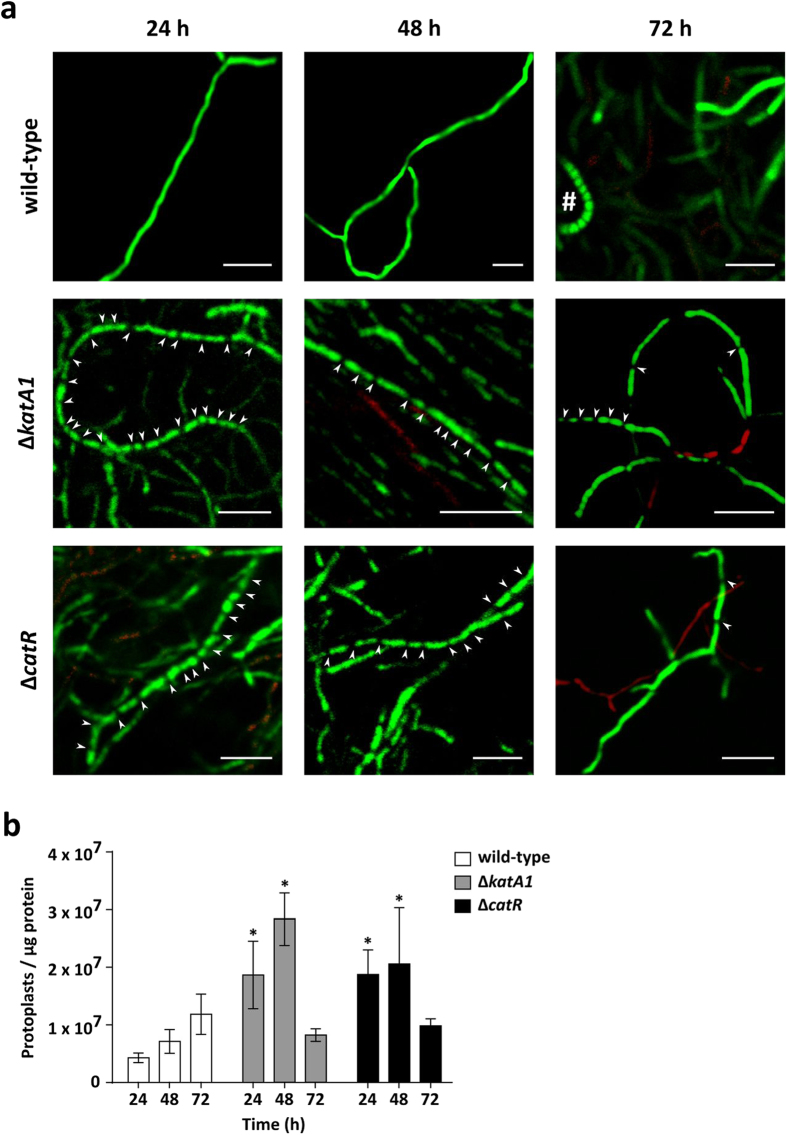
Assessment of the presence of compartmentalized (MI) and multinucleated (MII) mycelia in *S. natalensis* wild-type, Δ*katA1* and Δ*catR* strains. (**a**) Characterization of isolated hyphae through confocal microscopy using a cell viability assay. The presented micrographs are the result of merging the red (dead cells) and green (live cells) channels with ImageJ software. White arrowheads indicate the visible septa in the hyphae. # - aerial hypha forming a chain of spores. These results are representative of three independent experiments. Scale bar: 5 μm. (**b**) Quantification of protoplast formation through cytometry. Results are the average of biological duplicates and standard deviation. Statistically significant differences between strains at each time point were assessed by a one-way ANOVA followed by post hoc test (Tukey test; GraphPad Prism). * p < 0.01.

Morphological differentiation of the Δ*katA1* and Δ*catR* mutants and *S. natalensis* wild-type strain was characterized in solid cultures by confocal microscopy, making use of a live/dead cell specific staining protocol (Figs 5 and 6A).

At 24 h, the wild-type colonies were majorly composed by live hyphae that accumulated at the medium surface. Mycelium continued to proliferate and at 48 h the centre of the colony started to present cell death, while the most peripheral mycelium remained viable. At 72 h, colonies maintained this defined mycelium stratification, albeit the area occupied by dead mycelium was much larger than at 48 h (Fig. 5). This pattern showed that the development of streptomycetes colonies is a dynamic process where the “old hyphae” (colony centre) undergo PCD and the “young hyphae” (colony periphery) remain viable and differentiate. Detailed hyphae images of the wild-type (Fig. 6A) showed a low-septation pattern or absence of septa throughout the different time points, indicating a differentiation into the MII stage prior to 24 h. At 72 h spore chains were observed, showing that the wild-type is able to complete its development cycle in R5 medium.

Regarding Δ*katA1*, an accumulation of mycelium at the medium surface was also observed at 24 h. However, in the subsequent time points, mycelium presented an apparently independent growth above and within the medium: growth into the medium was restricted to small live hyphae aggregates, whereas the growth above the medium surface consisted in mycelium clumps, which were mostly alive, only presenting some cell death in the centre (Fig. 5). Hyphae of the mutant strain Δ*katA1* (Fig. 6A) showed a high-septation pattern at all time points (24 h, 48 and 72 h), demonstrating that the compartmentalized mycelium MI survives until later growth stages in this strain. Nevertheless, at 72 h some hyphae presented less frequent septa, pointing out to a transition from MI into MII (Fig. 6A).

As observed in the other strains, Δ*catR* presented an accumulation of mycelium at the medium surface at 24 h. Strikingly, Δ*catR* colonies showed a desynchronized behaviour at 48 h: the mycelium immersed in the medium presented cell death in the centre and live mycelium in the periphery, similar to the wild-type, whereas the mycelium located above the medium surface was mostly alive, lacking the stratification observed in the wild-type. At 72 h, the mycelium immersed in the medium degenerated; however, the mycelium grown above the medium-air interface was very heterogeneous: completely alive in some colonies, whereas others presented the wild-type dead/live stratification (Fig. 5). Observing hyphae in detail, Δ*catR* presented a high number of septa at 24 h and 48 h, demonstrating that its mycelium was in MI at these time points. At 72 h MII seemed to be fully formed, due to the predominance of low-septated hyphae and no MI was observed (Fig. 6A).

To quantify hyphae septation in *S. natalensis* mutant strains, we have measured the capacity of protoplasts formation in *S. natalensis* strains using the methodology developed recently by the research group of A. Manteca (unpublished results) (Fig. 6B). Manteca, *et al*.^27^ demonstrated that protoplast formation correlates with the septation of the mycelium, since MII multinucleated hyphae form big protoplast that are highly unstable, while MI compartmentalized hyphae form large numbers of stable protoplasts. The number of protoplasts generated by the mycelia from Δ*katA1* and Δ*catR* mutants at 24 h and 48 h was significantly higher than the wild-type. These results are consistent with the high-septation (MI) observed in the confocal micrographs of both mutant strains at these time points. At 72 h there were no significant differences between the mutants and the wild-type strain. Also interesting was the tendency of an increasing number of protoplasts formed by the wild-type mycelium throughout time. This happens also in other streptomycetes (Manteca *et al*. personal communication) and is consequence of sporulation, which led to an overestimation of the number of events counted by cytometry.

## Discussion

Streptomycetes morphological differentiation takes place in a pro-oxidant environment. Furthermore, it is well established that *Streptomyces* development program is triggered by physiological stresses^13^,^14^,^15^. Curiously, the construction of a *S. natalensis* knock-out mutant strain for an important antioxidant enzyme - the H_2_O_2_ inducible mono-functional catalase KatA1 - resulted in a blockage of aerial mycelium formation (bald phenotype) and to a complete lack of colony organization. In addition, deletion of its transcriptional repressor encoding gene *catR* resulted in a delayed but not completely impaired, aerial mycelium formation (Fig. 1). The lack of sporulation in both mutant strains hampered strain manipulation and consequently we were not able to perform mutant complementation. DNA delivery methodologies such as transformation or intergeneric conjugation using mycelium have been unfruitful for *S. natalensis*^28^. However, considering the genomic context of *catR* and *katA1* it is not likely that the mutations had a polar effect on the transcription of downstream genes.

Previously we have shown that KatA1 is the preferred antioxidant defence against H_2_O_2_-induced oxidative stress in *S. natalensis*^21^; the *katA1* expression profile and the induction of total catalase activity suggests that the onset of aerial mycelium is associated with H_2_O_2_ induced oxidative stress (Fig. 3). In addition, the expression of the bacterioferritin encoding gene *bfr* in the wild-type at 72 h emphasized the need to control the levels of free-iron in the cell, which is also a common response to oxidative stress^25^. The results suggest that the formation of aerial mycelium in *S. natalensis* is accompanied by the emergence of intracellular oxidative stress.

Given the important role played by KatA1 in the *S. natalensis* antioxidant background, it was not surprising to observe that the deletion of its encoding gene was accompanied with an adaptive response that comprised the activation of an array of defence mechanisms from early growth stages, namely the alternative mono-functional catalase KatA3, the ferritins Bfr and DpsB and the organic hydroperoperoxide resistance protein OhrB (Fig. 3; Table 1). The up-regulation of the iron-chelating ferritin encoding genes would likely lead to the decrease of intracellular iron availability in order to reduce iron-dependent H_2_O_2_ toxicity. Interestingly, it is known that the transcription of key development related genes in *S. coelicolor* is responsive to iron availability, in particular *bldN* transcription is down-regulated in iron limited conditions^29^. We hypothesize that the limited iron availability caused by the adaptive response to an early intracellular oxidative stress endured by Δ*katA1* mutant contributes to the down-regulation of *bldN* transcription and its regulatory cascade (chaplin and rodlin proteins), contributing to the observed bald phenotype. It should be also highlighted the induction of *dpsB* transcription (the only Dps encoding gene found in *S. natalensis* genome) in the Δ*katA1* mutant. Our results suggest that contrary to *S. coelicolor*^30^, *dpsB* transcription in *S. natalensis* might be induced by oxidative stress. Finally, it is noteworthy that despite the vast array of mechanisms that bacteria use to counteract redox imbalance, no proteins related with other redox systems (e.g. mycothiol) were identified by 2D-PAGE as presenting altered expression levels. However, for a complete characterization of the oxidative stress response a genome-wide approach would be needed.

Deletion of *catR* forced a constitutive over-expression of *katA1*. It would be safe to assume that the high levels of total catalase activity observed in Δ*catR* mutant prevent this strain to undergo H_2_O_2_ induced oxidative stress. Moreover, no major differences were observed in the transcription of ferritin encoding genes when compared to the wild-type strain. Nevertheless a down regulation of key development related genes is also observed in Δ*catR*. Although *katA1* is the only known target of CatR in *Streptomyces* sp.^16^,^21^, one cannot exclude the hypothesis that CatR orchestrates a wider molecular response, as the appearance of the blue-pigmented compound in Δ*catR* might suggest. In fact, the CatR homolog in *Bacillus subtilis* - PerR - was shown to coordinate the expression of several antioxidant defences encoding genes^31^.

Although no significant differences were observed in the transcription of the gene encoding the master regulator BldD, we cannot rule out its involvement in the arrest of morphological differentiation in both mutants. Cyclic di-GMP (c-di-GMP) is a bacterial second messenger that has been implicated in the regulation of numerous physiological processes, including oxygen sensing^32^ and the oxidative stress response^33^. Recently, it was also identified as a key mediator of BldD activity^7^. Thus, the intracellular redox status of the mutant strains might be modulating the cyclic di-GMP turnover and consequently BldD activity. Nevertheless, activation of the oxidative stress response machinery either by the generation of a sub-lethal oxidative stress (Δ*katA1*) or by de-repressing *katA1* expression (Δ*catR*), was associated with an active primary metabolism until later time points and with an increased vegetative mycelium proliferation rate. Moreover, the developmentally related PCD was shown to be inhibited (Δ*katA1*) or delayed (Δ*catR*) in the mutant strains, whereas in the wild-type, PCD was observed during aerial mycelium formation. Overall, these characteristics suggested an extended life-span of a mycelium in an early stage of development - the highly compartmentalized and transient mycelium MI.

The novelty of these observations, specifically regarding the prevalence of MI, required more conclusive data. The analysis of confocal micrographs showed that the wild-type was in the multinucleated stage MII at all time points (Fig. 6A). In addition the centre of the wild-type colonies was composed by dying hyphae that undergo PCD (Fig. 5), and aerial mycelium was being formed. It is noteworthy that this pattern followed the same trend observed in *S. coelicolor*^6^. Conversely, Δ*katA1* strain confocal micrographs and the analysis of the availability of these hyphae to form protoplasts, unequivocally demonstrated that a highly compartmentalized mycelium was present until 72 h, confirming the development arrest at MI. As a consequence, Δ*katA1* grew as a highly proliferative and undifferentiated mycelium with no defined colony organization (Fig. 1B and Fig. 5). Interestingly, this phenotype suggests that colony organization in the *S. natalensis* wild-type strain is dependent on the formation of the multinucleated mycelium MII. Regarding Δ*catR*, a differentiation blockage at MI was observed until 48 h. Nevertheless, at 72 h the great majority of the mycelium was already in MII (Fig. 6A), which was consistent with the appearance of colonies with the wild-type live/dead mycelium (Fig. 5) stratification and with the formation of a scarce aerial mycelium (Fig. 1B).

The delayed morphological differentiation of MI into MII displayed by Δ*katA1* and Δ*catR* mutant strains resembles the oxidative stress hormesis phenomenon described for other organisms, where an initial exposure to sub-lethal ROS dosages leads to the activation of an adaptive response and a concomitant increase in cell survival^34^. Ultimately, this hormetic effect implicates oxidative stress as a trigger of PCD, which has been previously demonstrated in *Escherichia coli*^35^. Taking into consideration this context, the phenotypes of Δ*katA1* and Δ*catR* strains demonstrate that the observed early activation of the oxidative stress response system avoided or delayed the first round of PCD, which blocked MI differentiation to MII. Importantly, this development arrest fully supports the mutant strains morphological phenotypes, since the emergence of MII is preceded by a PCD, and only MII can differentiate into aerial mycelium. In light of our results, we propose that the oxidative stress generated as a consequence of nutrient scarcity - main signal for the development program onset – and other physiological stresses is an important trigger of the developmentally related PCD process.

In conclusion, to the best of our knowledge, this work presents the first documented cases of *Streptomyces* strains arrested at the MI phase and show compelling evidences that place oxidative stress as an important player in the transition between developmental stages, namely in the differentiation of MI to MII. Additionally, it brings relevant knowledge for the industrial production of added-value compounds by *Streptomyces* strains, due to the strict correlation of MI/MII transition with the onset of secondary metabolism^36^.

## Methods

### *Streptomyces natalensis* genome sequencing

Whole-genome shotgun sequencing of *S. natalensis* strain ATCC 27448 was determined using an Illumina HiSeq 2000 sequencing system at BGI (www.genomics.cn). Two paired-end libraries of 0,5-kbp (10 000 000 reads) and 2-kbp (5 000 000 reads) were used for sequencing that yielded 900 and 450 Mbp of clean data, respectively. The reads were assembled using the SOAPdenovo software (version 1.05) of the SOAP tool package (http://soap.genomics.org.cn/soapdenovo.html). Scaffolds were submitted to GenBank as a whole shotgun sequence and annotated through the Prokaryotic Genome Annotation Pipeline (PGAP). This Whole Genome Shotgun project has been deposited at DDBJ/EMBL/GenBank under the accession JRKI00000000. The version described in this paper is JRKI01000000.

### Bacterial strains and growth conditions

*Escherichia coli* strains were routinely grown in LB medium at 37 °C. *S. natalensis* strains were routinely cultivated in TBO^37^ solid medium at 30 °C. Differentiation studies were performed in R5^38^ solid medium at 30 °C; 500 μl of YEME^39^ liquid cultures grown to late exponential phase were used as inoculum for *S. natalensis* solid cultures. To harvest mycelium for nucleic acids or protein extractions, *S. natalensis* strains were grown in a nitrocellulose membrane with a 0.45 μm pore (Merck Millipore) placed on top of R5 solid medium. For the mycelium proliferation assays, a drop of 10 μl of YEME liquid cultures grown to OD_600nm_ of 4–5 was inoculated in the centre of R5 plates. Plates were photographed and the mycelium area was determined using the ImageJ software.

### Construction of Δ*catR* knock-out mutant strain

The coding sequence of *catR* from *S. natalensis* ATCC 27448 was replaced by the *aac(3)IV/oriT* cassette from plasmid pIJ773 using a PCR-targeting approach^40^. The primers used for the amplification of the cassette were D_catR_F and D_catR_R (Supplementary Table S2). The native *catR* gene was replaced by the resistance cassette through homologous recombination resulting in the knock-out mutant strain *S. natalensis* Δ*catR::aac(3)IV/oriT*. The genetic identity of this strain was confirmed by Southern hybridization and PCR (Supplementary Fig. S1).

### Nucleic acids and protein procedures

*S. natalensis* genomic DNA was isolated from 1 ml of YEME liquid cultures using the MasterPure^TM^ Gram Positive DNA Purification Kit (Epicentre). Southern hybridization was performed with probes labelled with digoxigenin using the DIG DNA labelling kit (Roche). Intergeneric conjugation between *E. coli* ET12567 [pUZ8002] and *S. natalensis* ATCC 27448 was performed as previously described^28^.

For total RNA isolation from mycelium grown in solid medium, a portion of mycelium (approx. 2 cm^2^) was scrapped from the membrane and resuspended in 500 ml of K-phosphate buffer 50 mM pH 6.8. Two volumes of RNA Protect Bacteria Reagent (Qiagen) were added to the suspension; mycelium was harvested by centrifugation and immediately frozen by immersion in liquid nitrogen. RNA extraction was performed using RNeasy® Mini kit (Quiagen) and following the manufacturer instructions with modifications described elsewhere^21^. DNase treatments, RNA concentration determination and RNA quality and integrity evaluation was performed as previously described^21^.

Protein crude extracts were obtained from a section (approx. 2 cm^2^) of scrapped mycelium. This mycelium was washed and resuspended in the appropriate buffer containing 25% (v/v) of protease inhibitor (Roche). Mycelium disruption was performed through sonication (Branson Sonifier, Model B-15), the lysate was centrifuged and the pellet discarded. Protein content of cellular extracts was determined by the BCA^TM^ Protein Assay Kit (Pierce) or Bradford reagent (Bio-rad) when buffers contained reducing agents. Bovine serum albumin was used to determine standard curves. Unidimensional protein profiles were determined by SDS_PAGE in 12.5% (w/v) gels and using the coomassie coloration (Bio-Rad).

### Enzymatic activity assays

Catalase activity was quantified spectrophotometrically by following the rate of decrease in absorbance at 240 nm caused by the disappearance of H_2_O_2_^41^ as described elsewhere^21^. 1 unit of enzyme activity is defined as the amount required for the conversion of 1 μmol substrate into product per min. The specific activity of glyceraldeheyde 3-phosphate dehydrogenase (GAPDH) was measured as previously described^42^. 1 unit of GAPDH activity corresponds to the amount of enzyme necessary to produce 1 μmol of NADH per min, at 25 °C. The glucose 6-phosphate dehydrogenase (G6PDH) specific activity was determined spectrophotometrically by following the reduction rate of NADP^+^ into NADPH at 340 nm^43^. 1 unit of G6PDH activity is equivalent to the amount of enzyme necessary to produce 1 μmol of NADPH per min, at 25 °C. Aconitase specific activity was measured as previously described^44^. The rate of isocitrate formation from citrate was followed at 240 nm, using an absorption coefficient of 3.6 mM^−1^. 1 unit of activity transformed 1 nmol of substrate per minute, at 25 °C.

In gel determination of catalase activity was performed in 7.5% (w/v) non-denaturing polyacrylamide gels as previously described^45^. For nuclease activity, protein extracts were separated in 12.5% (w/v) polyacrylamide gels in denaturing conditions containing DNA from herring testes type XIV (SIGMA) 10 μg/ml. Protein renaturation, induction of nuclease activity and gel visualization were performed as previously described^46^.

### Two-dimensional polyacrylamide gel electrophoresis

The two-dimensional polyacrylamide gel electrophoresis (2D-PAGE) technique was performed as previously described^47^. Total protein extracts, isoelectric focusing and SDS-PAGE gels were prepared as described elsewhere^21^. Gels were silver stained through a mass spectrometry (MS) compatible protocol^48^. PageRuler^TM^ (Fermentas) was used as size marker. *In silico* analysis of the 2D gels was performed in PDQuest 2-D (Bio-Rad). Spots with a significant statistical difference between strains (biological duplicates) were considered using the following criteria: *P* > 0.05 and two-fold change.

Protein spots were excised from gels and digested with trypsin. Samples were analysed using the 4700 Proteomics Analyzer MALDI-TOF/TOF (Applied Biosystems) as previously described^49^. Data were analysed using GPS Explorer (Version 3.6; Applied Biosystems). Protein identification was performed combining data from PMF (Peptide Mass Fingerprint) and tandem mass (MS/MS) spectra. The Mascot (Matrix Science, UK) algorithm was used to determine the statistical significance (score > 52) of protein identification against the genome database of *S. natalensis* ATCC 27448.

### PCR and RT-PCR

DNA amplification by PCR was performed with GoTaq Flexi DNA Polymerase (Promega) or Pfu DNA polymerase (Fermentas) according to manufacturer instructions.

For gene expression studies 1 μg of DNase I-treated (DNA-free Kit, Ambion) total RNA was transcribed with the iScript^TM^ Select cDNA Synthesis Kit (Bio-Rad), using the random primers supplied, and following the manufacturer instructions. RT-PCR amplifications were performed using the primer pairs listed in Supplementary Table S2 and using GoTaq Flexi DNA Polymerase (Promega). PCR products were separated on 2% (w/v) agarose gels.

### Confocal microscopy

To prepare samples for confocal microscopy analysis, 4 cm^2^ blocks of R5 solid cultures were cut out using a scalpel and sliced using a microtome (sections with a width of approx. 0.15 mm). To characterize isolated hyphae, mycelium was scrapped from R5 solid cultures and resuspended in NaCl 0,9% (w/v). 10 μl of the mycelium resuspension were used for staining. Viability staining was performed using the “LIVE/DEAD *Bac*Light Bacterial Viability Kit” (Molecular Probes) with a mixture of SYTO9 and propidium iodide in a 1:1 proportion and following the manufacturer instructions. Samples were observed under a Leica SP2 AOBS SE confocal laser-scanning microscope using 488 nm and 568 nm as excitation wavelengths and emitting between 520–545 nm (green) or 620–640 nm (red), as previously described^6^. Micrographs were treated using the software ImageJ.

### Mycelium protoplasting and protoplast quantification

Protoplast were obtained and quantified according to the methodology developed recently by Manteca *et al*. (unpublished results). These authors made the control experiments necessary to warrant that the protocol used for protoplast formation had an efficiency close to 100%, which is necessary for reproducibility of the results, and to warrant the correlation between hypha compartmentalization and protoplast formation. Briefly, 60 mg of mycelia (fresh weight) were resuspended in 1.4 ml of modified buffer P (0.6% TES buffer pH 7.2, 103% sucrose; filtered through a 0.2 μm filter) into a 2-ml Eppendorf tube. Lysozyme was added from a freshly prepared stock at a final concentration of 2 mg/ml and incubated for 30 minutes at 600 rpm and 37 °C. Protoplasts were drawn in and out in a 1-ml pipette two times, incubated for an additional 30 minutes, washed two times by sedimentation (1000 g) and resuspended in buffer P. After the last washing step, protoplasts were resuspended in 500 μl of buffer P.

Samples were stained with SYTO9 (Invitrogen, 6 μM) and quantified with a flow cytometer (Cytomics FC500, Beckman-Coulter Inc., USA). Measurements were performed using BD Trucount Tubes®, with 500 μl of protoplasts stained with SYTO9. The trigger signal was established with an FL1 detector (530/540 nm) with the adequate negative control (buffer P) and biological control (BD Plasma Count®). The absolute quantifications were performed counting 10,000 of the standard beads included in the BD Trucount Tubes®. Protoplast dilutions used for cytometry quantifications were those reporting absolute protoplast numbers within the 5,000–10,000 interval, close to the number of beads used as standard. The number of protoplasts per μl was calculated based on the number of standard beads, and the number of protoplasts per mg of protein was calculated based on the protein estimated for the mycelium processed for protoplast formation. Protoplasts from two biological replicates were quantified.

## Additional Information

**How to cite this article**: Beites, T. *et al*. *Streptomyces natalensis* programmed cell death and morphological differentiation are dependent on oxidative stress. *Sci. Rep*. **5**, 12887; doi: 10.1038/srep12887 (2015).

## Supplementary Material

Supplementary Information

## Figures and Tables

**Table 1:** List of identified proteins in the 2D-PAGE proteome comparison between the *S. natalensis* mutant strains ∆*katA1* and ∆*catR* with the wild-type.

SNA code (spot ID§)	Description	Δ*katA1* vs wt (fold change)	Δ*catR* vs wt (fold change)
**Ribosome constituents**			
SNA_38815 (4)	50S ribosomal protein L7/L12	Up (*)	Absent
**Chaperones**			
SNA_03365 (6)	molecular chaperone DnaK	Down (0.5)	Down (# 0.5)
**Protection responses**			
SNA_07730 (11)	organic hydroperoxide resistance reductase B	Up (*)	Up (*)
SNA_31980 (37)	recombinase A	Up (*)	Absent
SNA_34325 (28)	catalase	Absent	Up (27)
**Macromolecule degradation**			
SNA_02830 (39)	aminopeptidase	Up (*)	Absent
SNA_12425 (34)	serine protease 2	Absent	Up (*)
SNA_32135 (30)	RNA-metabolising metallo-beta-lactamase	Up (*)	Absent
SNA_32175 (10)	guanosine pentaphosphate synthetase/polyribonucleotide nucleotidyltransferase	Absent	Absent
**Aminoacid biosynthesis**			
SNA_33095 (21)	acetolactate synthase	Up (8.8)	Absent
**Biosynthesis of cofactors, carriers**			
SNA_36475 (24)	bifunctional 5,10-methylene-tetrahydrofolate dehydrogenase/5,10-methylene-tetrahydrofolate cyclohydrolase	Up (3.8)	Down (# 0.4)
**Central intermediary metabolism**			
SNA_09155 (8)	ribokinase	Down (0.4)	Down (# 0.7)
**Degradation of small molecules**			
SNA_17045 (40)	glyoxylate carboligase	Up (*)	Absent
SNA_33060 (12)	1-pyrroline-5-carboxylate dehydrogenase	Down (0.3)	Down (0.2)
**Energy metabolism, carbon**			
SNA_07865 (33)	pyruvate dehydrogenase E1	Up (*)	Absent
SNA_09035 (35)	dihydrolipoamide acyltransferase	Absent	Up (*)
SNA_09040 (27)	dihydrolipoamide dehydrogenase	Up (*)	Up (*)
SNA_10650 (22)	glucose 6-phosphate dehydrogenase	Up (*)	Absent
SNA_12565 (25)	alanine dehydrogenase	Up (*)	Absent
SNA_33550 (19)	pyruvate kinase	Up (5.7)	Up (# 1.5)
**Nucleotide biosynthesis**			
SNA_12580 (31)	CTP synthetase	Up (*)	Absent
SNA_13390 (38)	carbamoyl phosphate synthase small subunit	Up (*)	Absent
SNA_36490 (18)	purine biosynthesis protein	Up (*)	Absent
**Unknown function**			
SNA_01165 (17)	Hypothetical protein	Up (*)	Up (*)
SNA_17125 (36)	superfamily DUF2263	Up (# 3.8)	Up (5.6)
SNA_27255 (23)	Hypothetical protein	Down (# 0.7)	Down (0.4)

Proteins were grouped according to the functional categorization available for *S. coelicolor* genome (ftp://ftp.sanger.ac.uk/pub/ project/pathogens/S_coelicolor).

^*^Not detected in the wild-type.

^#^Not statistically significant different (*P* < 0.05).

^§^Spot ID refers to protein spot numbers in Supplementary Fig. S2.
