# Supplementary material for: Streptomyces natalensis programmed cell death and morphological differentiation are dependent on oxidative stress
Source: Sci Rep. 2015 Aug 10;5:12887. doi: 10.1038/srep12887 (PMC4530454; doi:10.1038/srep12887)
Supplement: Supplementary Information [file srep12887-s1.pdf]

# Supplementary Information

**Streptomyces natalensis programmed cell death and morphological differentiation are dependent on oxidative stress**

**Tiago Beites[1,2], Paulo Oliveira[1,2], Beatriz Rioseras[3], Sílvia D.S. Pires[1,2,4], Rute Oliveira[1,2], Paula Tamagnini[1,2,5], Pedro Moradas-Ferreira[1,2,4], Ángel Manteca[3] and Marta V. Mendes[1, 2]***

1 – i3S - Instituto de Investigação e Inovação em Saúde, Universidade do Porto, Porto, Portugal.

2 - IBMC – Instituto de Biologia Molecular e Celular, Universidade do Porto, Porto, Portugal.

3 - Área de Microbiología, Departamento de Biología Funcional e IUOPA, Facultad de Medicina, Universidad de Oviedo, Oviedo, Spain.

4 - ICBAS – Instituto de Ciências Biomédicas Abel Salazar, Universidade do Porto, Porto, Portugal.

5 - Faculdade de Ciências, Departamento de Biologia, Universidade do Porto, Porto, Portugal.

**Supplementary Table S1** – *S. natalensis* homologues.

**Supplementary Table S2** – Primers.

**Supplementary Figure S1** - Construction of *S. natalensis* Δ*catR* strain by gene replacement.

**Supplementary Figure S2** – 2D-PAGE gels.

**Supplementary Figure S3** – SDS-PAGE.

**Table S1.** *S. natalensis* homologues of known *S. coelicolor* development related proteins.

| Protein name | SNA_code | Identity | Coverage | e-value | *S. coelicolor* RefSeq |
|---|---|---|---|---|---|
| **BldA** | SNA_04225 | 93% | 100% | 4.00E-28 | Y00209.1 |
| **BldB** | SNA_08815 | 68% | 93% | 1.00E-23 | NP_629849.1 |
| **BldC** | SNA_03650 | 100% | 100% | 6.00E-44 | NP_628272.1 |
| **BldD** | SNA_13365 | 98% | 100% | 4.00E-113 | NP_625769.1 |
| **BldG** | SNA_00365 | 96% | 100% | 1.00E-73 | NP_627747.1 |
| **BldH (AdpA)** | SNA_06025 | 92% | 79% | 0 | NP_627022.1 |
| **BldKA** | SNA_34770 | 60% | 99% | 8.00E-146 | NP_629262.1 |
| **BldKB** | SNA_34775 | 49% | 97% | 0 | NP_629263.1 |
| **BldKC** | SNA_34780 | 70% | 99% | 5.00E-166 | NP_629264.1 |
| **BldKD** | SNA_34785 | 78% | 99% | 0 | NP_629265.1 |
| **BldKE** | SNA_34790 | 80% | 93% | 0 | NP_733655.1 |
| **BldM** | SNA_39180 | 100% | 100% | 7.00E-142 | NP_628926.1 |
| **BldN** | SNA_38050 | 96% | 67% | 7.00E-123 | NP_627533.1 |
| **WhiA** | SNA_10605 | 92% | 99% | 0 | NP_626214.1 |
| **WhiB** | SNA_03895 | 95% | 98% | 7.00E-56 | NP_627256.1 |
| **WhiD** | SNA_39175 | 97% | 84% | 4.00E-64 | NP_628925.1 |
| **WhiEI** | SNA_26210 | 57% | 96% | 2.00E-138 | NP_629462.1 |
| **WhiEII** | SNA_26205 | 72% | 91% | 9.00E-65 | NP_629461.1 |
| **WhiEIII** | SNA_26200 | 76% | 99% | 0 | NP_629460.1 |
| **WhiEIV** | SNA_26195 | 74% | 96% | 0 | NP_629459.1 |
| **WhiEV** | SNA_26190 | 52% | 98% | 9.00E-23 | NP_629458.1 |
| **WhiEVI** | SNA_26185 | 68% | 91% | 7.00E-69 | NP_629457.1 |
| **WhiEVII** | SNA_26180 | 72% | 95% | 2.00E-52 | NP_629456.1 |
| **WhiEVIII** | SNA_26175 | 77% | 96% | 0 | NP_629463.1 |
| **WhiG** | SNA_32585 | 90% | 99% | 0 | NP_629755.1 |
| **WhiH** | SNA_31720 | 76% | 99% | 7.00E-162 | NP_629942.1 |
| **WhiI** | SNA_30685 | 95% | 99% | 9.00E-140 | NP_630140.1 |
| **ChpA** | SNA_06310 | 50% | 56% | 3.00E-37 | NP_733581.1 |
| **ChpC** | SNA_01235 | 46% | 99% | 5.00E-38 | NP_625949.1 |
| **ChpD** | SNA_00490 | 67% | 97% | 2.00E-23 | NP_626950.1 |
| **ChpD2** | SNA_12255 | 77% | 60% | 1.00E-20 | NP_626950.1 |

| Protein name | SNA_code | Identity | Coverage | e-value | *S. coelicolor* RefSeq |
|---|---|---|---|---|---|
| **ChpE** | SNA_11245 | 64% | 97% | 6.00E-20 | NP_626070.1 |
| **ChpE2** | SNA_11250 | 67% | 64% | 1.00E-17 | NP_626070.1 |
| **ChpG** | SNA_00485 | 67% | 49% | 2.00E-16 | NP_626939.1 |
| **ChpG2** | SNA_05975 | 73% | 48% | 3.00E-15 | NP_626939.1 |
| **ChpH** | SNA_12040 | 86% | 98% | 8.00E-24 | NP_625950.1 |
| **RdlA** | SNA_27905 | 53% | 71% | 2.00E-25 | NP_626951.1 |
| **RamR** | SNA_20600 | 50% | 98% | 1.00E-50 | NP_733711.1 |
| **RamB** | SNA_20605 | 56% | 96% | 1.00E-174 | NP_630759.1 |
| **RamA** | SNA_20610 | 56% | 92% | 8.00E-147 | NP_630758.1 |
| **RamS** | SNA_20615 | 76% | 97% | 3.00E-17 | NP_630757.1 |
| **RamC** | SNA_20620 | 66% | 99% | 0 | NP_630756.1 |
| **DivIVA** | SNA_09545 | 84 % | 54 % | 4E-99 | NP_626336 |
| **FilP** | SNA_33665 | 92% | 100 % | 0 | NP_629535 |
| **FtsK** | SNA_11150 | 61 % | 88 % | 1E-152 | NP_733622 |
| **FtsW** | SNA_09505 | 79 % | 94% | 0 | NP_626344 |
| **FtsZ** | SNA_09520 | 86 % | 100 % | 0 | NP_626341 |
| **Mbl** | SNA_06725 | 97 % | 100 % | 0 | NP_626848 |
| **MreB** | SNA_07640 | 71 % | 98 % | 4E-170 | NP_626694 |
| **ParA** | SNA_02365 | 89 % | 100 % | 4E-173 | NP_628072 |
| **ParB** | SNA_02360 | 74 % | 99 % | 1E-149 | NP_628073 |
| **Scy** | SNA_33660 | 60 % | 100 % | 0 | NP_629536 |
| **SigF** | SNA_01565 | 92 % | 94 % | 8E-171 | NP_628217 |
| **Smc** | SNA_32725 | 82 % | 86 % | 0 | NP_629712 |
| **SsgA** | SNA_26640 | 49 % | 97 % | 4E-40 | NP_630795 |
| **SsgB** | SNA_00325 | 88 % | 92 % | 2E-159 | NP_627756 |
| **SspA** | SNA_27035 | 44 % | 61 % | 3E-3 | NP_631483 |

**Table S2.** Primers used in PCR and RT-qPCR.

| Primer | Sequence | T.A. (ºC) | Amplicon (bp) |
|---|---|---|---|
| **D_catR_F** | ATTCCGGGGATCCGTCGACCGTACCCTGGGCTGTATCTGACTGATAGGTGATTGGCATG | 60 | 1462 |
| **D_catR_R** | TGTAGGCTGGAGCTGCTTCCCTGGGGGCGGGCCGGACGCGGGACGCCGACCGCGGTCA | | |
| **C_catR_F** | GTGCGCCTCCTGAAGTGATC | 60 | 584 |
| **C_catR_R** | TCAGGCGTGGTGGCCGCT | | |
| **adpA_S** | CCCGCGACCACCCATTGGA | 65 | 105 |
| **adpA_AS** | GGTGAGCACATCGCCGTCGTC | | |
| **bldD_S** | CTGGAAGGCCGTGGTGGTGG | 65 | 105 |
| **bldD_AS** | AGCTCCTGGACCGGCACCC | | |
| **bldN_S** | CACGGCCGAGGACCTCACCA | 65 | 102 |
| **bldN_AS** | ATGGTCACCAGCCAGGCGC | | |
| **chpC_S** | CACCAGGGCGGGTCGGA | 60 | 109 |
| **chpC_AS** | TGGCCGGTACCGCGTAGC | | |
| **ramC_S** | ATCCATATCTCGGCCTGCCTGG | 60 | 102 |
| **ramC_AS** | CCGGCTCGGCATGCATTTG | | |
| **ramS_S** | TCGACCTGCAGACGATGGAAAC | 60 | 99 |
| **ramS_AS** | TCAGACTGCTGTCGCCGCA | | |
| **rdlA_S** | GAGTAACCGCCACGCCAGCA | 60 | 103 |
| **rdlA_AS** | CCCTGCGGGCTCATGTCG | | |
| **bfrA_S** | CGGCTGGCCGAAGCTTGC | 60 | 103 |
| **bfrA_AS** | CGGCAGCCCTTCGAGGAAGA | | |
| **catR_S** | CAACGCCCACCACGACCA | 65 | 75 |
| **catR_AS** | TCGCCCTGGAGATGGACG | | |
| **cpx_S** | GAGCGGCTCCAGCAGGACT | 60 | 106 |

| Primer | Sequence | T.A. (ºC) | Amplicon (bp) |
|---|---|---|---|
| **cpx_AS** | TCGCGGCTTGTTCGACG | | |
| **dpsA_S** | CACTGGAACGTCGTCGGCC | 60 | 150 |
| **dps_AS** | CTTGGCGACCGTCGAGGC | | |
| **katA1_S** | CCTGGTCGGTCTTGAAGTGGTA | 60 | 80 |
| **katA1_AS** | CTACGGCTCGCACACCTTC | | |
| **katA3_S** | CACACCAAGGACGACGACTT | 60 | 81 |
| **katA3_AS** | CTCCACCAGACGCTTCTTCTC | | |
| **16S_S** | CAGGCTAGAGTTCGGTAGG | 65 | 117 |
| **16S_AS** | CTCCTCAGCGTCAGTATCG | | |

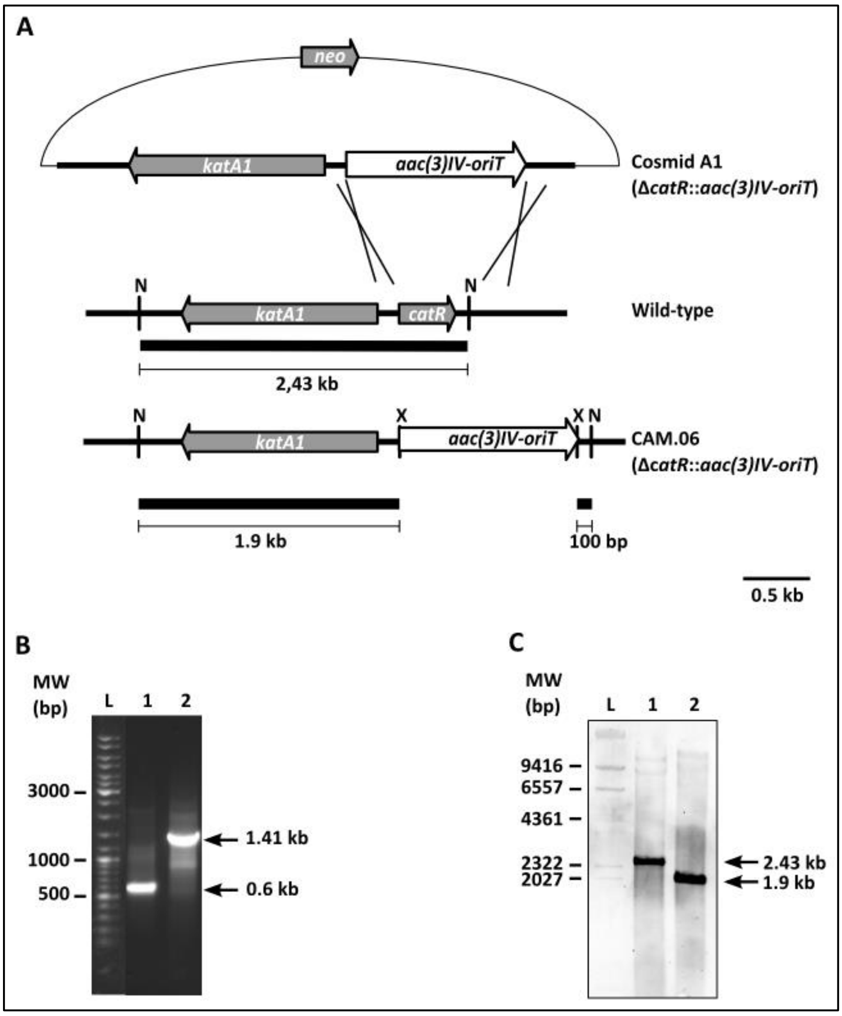


**Fig. S1 - Construction of *S. natalensis* Δ*catR* strain by gene replacement.**

(A) Predicted restriction enzyme polymorphism caused by gene replacement. The *Nco*I-*Xba*I restriction pattern before and after replacement is shown. The probe used for southern hybridization is indicated by thick lines. N, NcoI; X, XbaI. (B) Confirmation of gene disruption by PCR. A pair of primers, C_catR_F and C_catR_R, covering the deleted region in the chromosome were to identify double crossover mutants. Lane L, GeneRuler$^{TM}$ (Thermo Scientific).(C) Confirmation of gene disruption by Southern blot of the NcoI-XbaI digested chromosomal DNA of the wild type (lane 1), and Δ*catR* (lane 2) strains. Lane L, DIG-labelled DNA Molecular Weight Marker II (Roche).

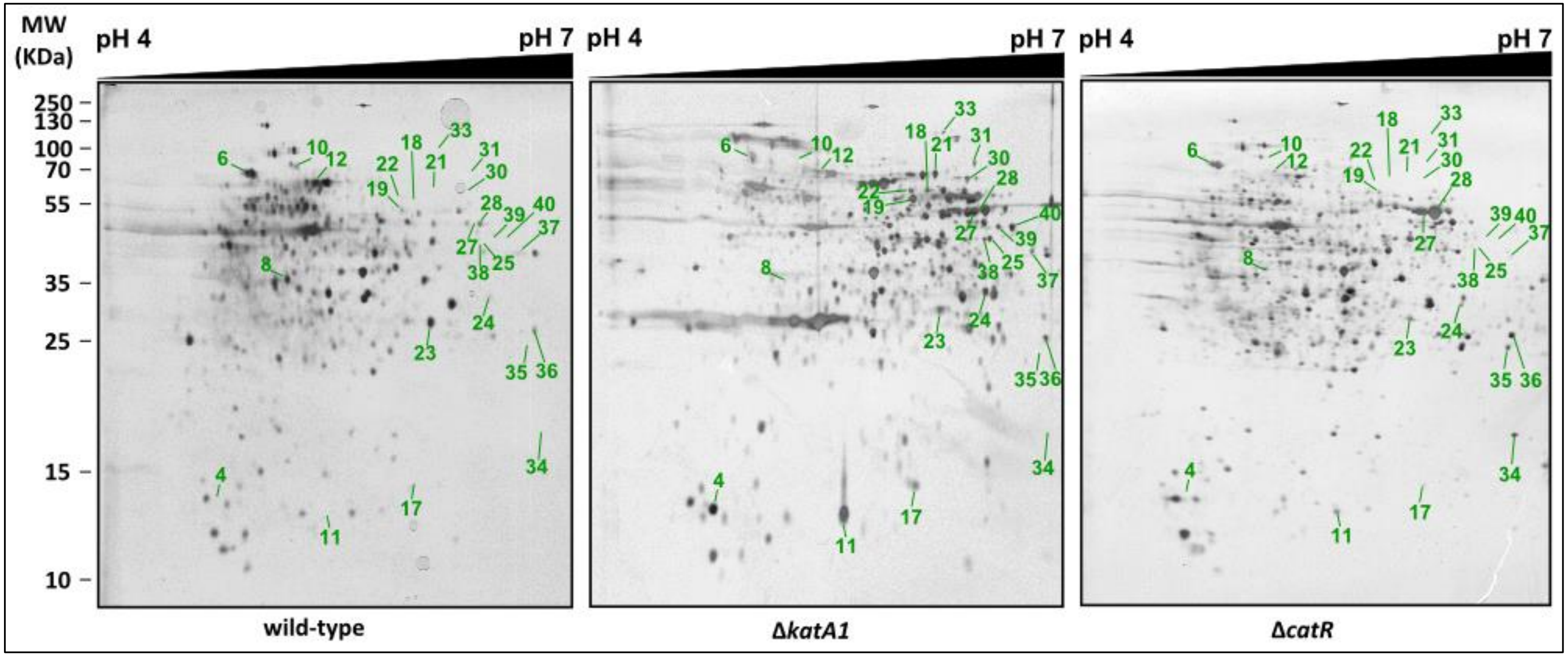


**Fig. S2 - Proteome analysis by 2D-PAGE**.

The presented 2D gels are representative of biological duplicates (100 μg of crude protein extracts per gel). The spots indicated in the figure correspond to the proteins identified by mass spectrometry (for SNA code correspondence to spot ID number see Table 1).

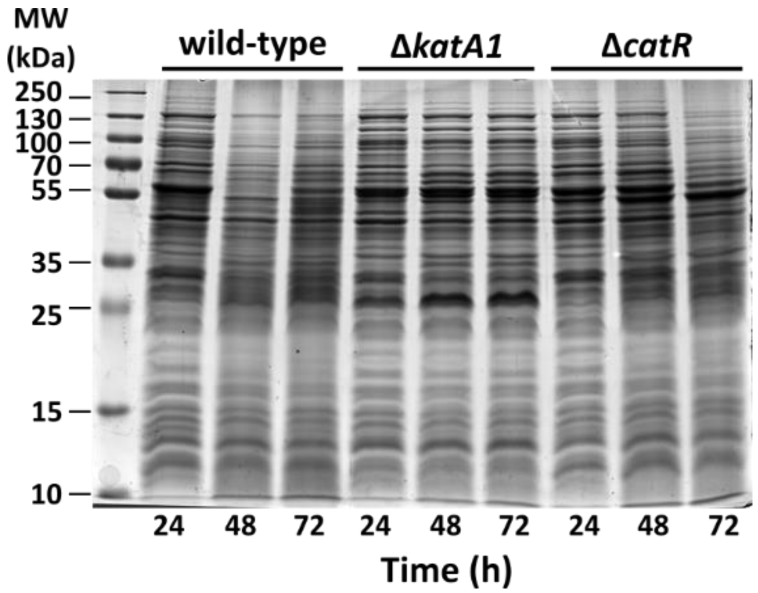


**Fig. S3 -** Coomassie coloration of SDS-PAGE with protein crude extracts (20 μg of protein per lane) used as loading control for the nuclease activity assay (Fig. 4B).
